# Vitamin D, Gut Microbiota, and Cancer Immunotherapy—A Potentially Effective Crosstalk

**DOI:** 10.3390/ijms26157052

**Published:** 2025-07-22

**Authors:** Yizhen Yan, Yi Guo, Yiting Li, Qingrui Jiang, Chenhang Yuan, Li Zhao, Shanshan Mao

**Affiliations:** 1 Beijing Key Laboratory of Sports Performance and Skill Assessment, Beijing Sport University, Beijing 100084, China; yanyiz@126.com (Y.Y.);; 2 School of Sports Medicine and Rehabilitation, Beijing Sport University, Beijing 100084, China; yi___guo@163.com (Y.G.);

**Keywords:** vitamin D, gut microbiota, cancer immunotherapy, crosstalk

## Abstract

Recent breakthroughs in cancer immunotherapy have shown remarkable success, yet treatment efficacy varies significantly among individuals. Emerging evidence highlights the gut microbiota as a key modulator of immunotherapy response, while vitamin D (VD), an immunomodulatory hormone, has garnered increasing attention for its potential interactions with gut microbiota and immunotherapy outcomes. However, the precise mechanisms and clinical applications of VD in this context remain controversial. This study systematically analyzed peer-reviewed evidence from PubMed, Scopus, Web of Science, PsycINFO, and MEDLINE (January 2000–May 2025) to investigate the complex interplay among VD, gut microbiota, and cancer immunotherapy. This review demonstrates that VD exerts dual immunomodulatory effects by directly activating immune cells through vitamin D receptor (VDR) signaling while simultaneously reshaping gut microbial composition to enhance antitumor immunity. Clinical data reveal paradoxical outcomes: optimal VD levels correlate with improved immunotherapy responses and reduced toxicity in some studies yet are associated with immunosuppression and poorer survival in others. The bidirectional VD–microbiota interaction further complicates this relationship: VD supplementation enriches beneficial bacteria, which reciprocally regulate VD metabolism and amplify immune responses, whereas excessive VD intake may disrupt this balance, leading to dysbiosis and compromised therapeutic efficacy. These findings underscore the need to elucidate VD’s dose-dependent and microbiota-mediated mechanisms to optimize its clinical application in immunotherapy regimens. Future research should prioritize mechanistic studies of VD’s immunoregulatory pathways, personalized strategies accounting for host–microbiota variability, and large-scale clinical trials to validate VD’s role as an adjuvant in precision immunotherapy.

## 1. Introduction

Cancer immunotherapy has revolutionized oncology by harnessing the immune system to target and eliminate malignant cells. Despite its transformative potential, the efficacy of immunotherapy varies widely among patients, with many experiencing limited responses or significant adverse effects. This variability underscores the need to identify factors that can optimize treatment outcomes [1,2].

Among these, vitamin D (VD) and the gut microbiota have emerged as critical modulators of immune function, with growing evidence suggesting their interplay may influence immunotherapy efficacy. VD, traditionally recognized for its role in calcium homeostasis and bone health, also exhibits potent immunomodulatory properties. It acts through the vitamin D receptor (VDR), which is expressed on immune cells, regulating their differentiation, activation, and cytokine production [3,4,5,6]. Recent studies have highlighted VD’s ability to shape the gut microbiota, promoting the growth of beneficial bacteria such as *Bacteroides fragilis*, which, in turn, enhances antitumor immunity [7]. Conversely, VD deficiency or excess can disrupt immune balance, leading to paradoxical effects such as immunosuppression or heightened inflammation [8,9].

The gut microbiota, a dynamic ecosystem of microorganisms, plays a pivotal role in immune regulation and has been linked to immunotherapy responses. Specific microbial taxa, including *Akkermansia muciniphila* and *Bifidobacterium*, are associated with improved outcomes by modulating immune cell activity and the tumor microenvironment [10,11,12,13,14,15,16]. Notably, VD and the gut microbiota engage in bidirectional interactions: VD influences microbial composition, while gut bacteria metabolize VD into its active form, creating a feedback loop that impacts host immunity [17,18].

This review examines the tripartite relationship among VD, gut microbiota, and cancer immunotherapy, synthesizing current evidence on their synergistic and context-dependent effects. It further analyzes the mechanisms by which VD and microbiota regulate immune responses, reviews clinical findings—including conflicting evidence on VD’s role in immunotherapy—and explores future research directions and therapeutic applications. By elucidating these interactions, this review aims to provide novel insights into leveraging VD and microbiota modulation to enhance immunotherapy efficacy and develop personalized cancer treatment strategies.

## 2. Methods

### 2.1. Search Strategy

**Figure 1:**
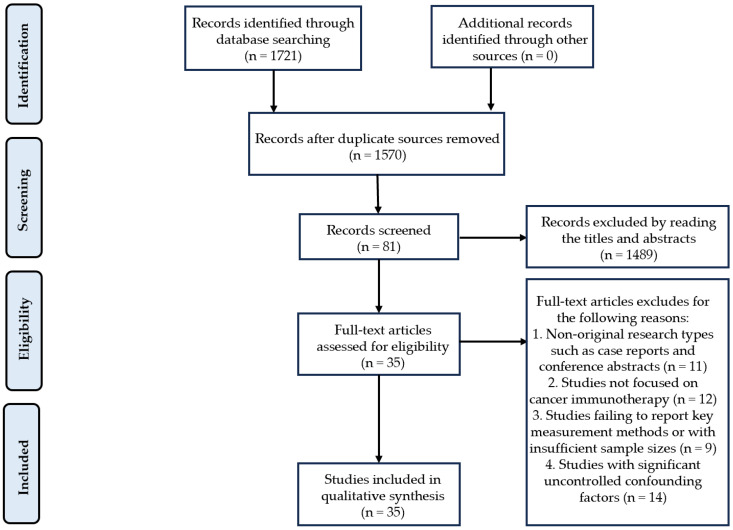
PRISMA flowchart for the selection of studies.

This review was conducted in PubMed, Scopus, Web of Science, PsycINFO, and MEDLINE, covering publications from January 2000 to May 2025. Medical subject headline terms included “Vitamin D and Immunotherapy”, “Vitamin D and Gut Microbiota”, “Gut Microbiota and Immunotherapy”, and “Vitamin D and Gut Microbiota and Immunotherapy”. We explored our proposed association by examining diverse levels of evidence, as discussed successively (Figure 1).

### 2.2. Inclusion Criteria

(a) Study types include randomized controlled trials, cohort studies, case–control studies, cross-sectional studies, and systematic reviews. Animal or cellular experimental studies with clearly defined mechanistic investigations are also eligible;
(b) For human studies, participants must be cancer patients receiving immunotherapy. Animal studies must examine the effects of VD or gut microbiota on immunotherapy outcomes;
(c) Studies must explicitly report VD status or gut microbiota composition and their interactions, with particular focus on the bidirectional regulatory relationship between VD and gut microbiota;
(d) Primary outcomes include immunotherapy response rates and survival data. Secondary outcomes encompass immune-related adverse events and changes in microbiota diversity.

### 2.3. Exclusion Criteria

(a) Non-original research types such as case reports and conference abstracts will be excluded;
(b) Studies not focused on cancer immunotherapy will be excluded;
(c) Studies failing to report key measurement methods or with insufficient sample sizes will be excluded based on data quality considerations;
(d) Studies with significant uncontrolled confounding factors will be excluded;
(e) Duplicate publications or studies irrelevant to the research topic will not be considered.

### 2.4. Quality Assessment

This systematic review employed standardized assessment tools tailored to different study types to evaluate the methodological quality of the included literature comprehensively. Systematic reviews were assessed using the AMSTAR checklist (11 items, total score of 0–11), with scores ranging from 0–4 indicating low quality, 5–8 indicating moderate quality, and 9–11 indicating high quality (Table 1). Observational studies were evaluated using the Newcastle-Ottawa Scale (NOS) across three domains (selection of study participants, comparability among groups, and outcome assessment), with a maximum score of 9 stars, as follows: 1–3 stars for low quality, 4–6 for moderate quality, and 7–9 for high quality (Table 2). Randomized controlled trials were assessed using the modified Jadad scale (0–7 points), where scores of 1–3 indicated low quality and scores of 4–7 indicated high quality (Table 3). Preclinical animal studies were evaluated using the Systematic Yardstick for Evaluating Credibility of Laboratory Experimental Studies (SYECLE) tool for bias risk assessment (Table 4). Although some items were rated as unclear in certain studies, their methodological credibility was still acknowledged, given their publication in high-impact-factor journals. This multi-tool approach ensured a rigorous and appropriate quality assessment for each study design included in the analysis.

The NOS criteria consist of three main sections:

(a) Selection of Study Groups:
  - Representativeness of the exposed cohort (★): Rated from “fully representative of population” to “not described” (4 tiers).
  - Selection of non-exposed group (★): Highest score if drawn from the same community; lower if from different sources/not described.
  - Ascertainment of exposure (★): Priority given to secure records or structured interviews; self-reports or no description score lower.
  - Absence of outcome at baseline (★): Must confirm no pre-existing outcome (“yes” scores).
(b) Comparability:
  - Control for the most important confounder (★), with additional control for other factors (★).
(c) Outcome Assessment:
  - Evaluation method (★): Blind independent assessment or record linkage scores highest; self-reports or no description score lower.
  - Follow-up duration (★): Must be sufficient and predefined.
  - Follow-up adequacy (★): Complete follow-up or low-bias attrition (e.g., <20% loss) scores highest.

## 3. VD Metabolism and Function

VD deficiency is a major public health problem for all ages worldwide, even in countries with perennial sun exposure [44,45,46]. The main source of VD in humans (90%) is the transformation of 7-dehydrocholesterol, which is developed in the skin after UVB radiation from the sun, into preVD. Only 10% of VD is obtained through dietary intake. VD is a fat-soluble vitamin in two forms, VD2 (ergocalciferol) and VD3 (cholecalciferol), both available through the diet. Vitamins D2 and D3 are transformed into 25-hydroxyVD (25(OH)D), by 25-hydroxylase enzymes such as CYP27A1 and CYP2R1 in the liver, which is the main circulating form of VD. Subsequently it is hydroxylated to 1,25 dihydroxyVD (1,25(OH)2D) in the kidneys by the 25(OH)D-1α-hydroxylase CYP27B1 [3,47].

**Figure 2:**
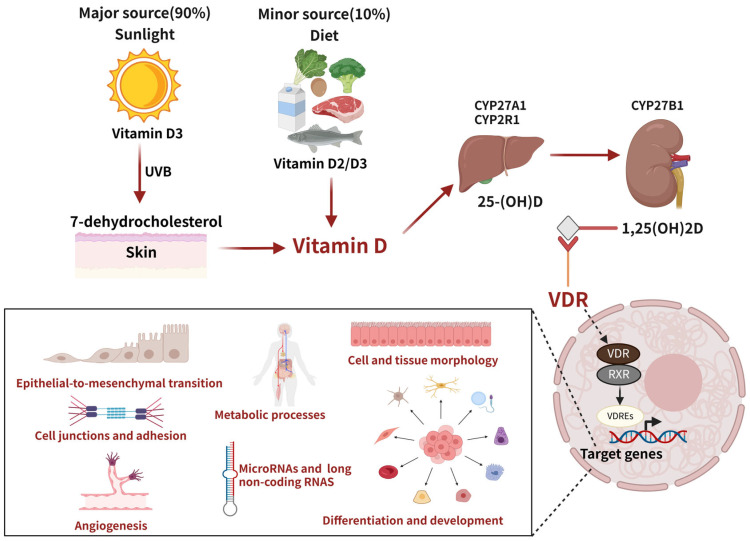
Vitamin D (VD) metabolism and mechanism of action. The circulating form of VD in the blood and its mechanism of action through vitamin D receptors (VDRs). A VDR is a nuclear hormone receptor and transcription factor present in almost all cell types. In the nucleus, a VDR forms a heterodimer with the retinoid X receptor (RXR). This enables the VDR/RXR complex to bind to vitamin D response elements (VDREs) in target genes and regulate their transcription, as well as the aspects encompassed by the target genes.

1,25(OH)2D served as the primary ligand of VDR. The VDR is a nuclear hormone receptor and transcription factor in virtually all cell types. The VDR forms a heterodimer within the nucleus with the retinoid X receptor (RXR). This enables the VDR/RXR complex to bind to VD-responsive elements (VDREs) in target genes and regulate their transcription. As a result, VD modulates numerous cellular processes, with one of its most significant effects being the regulation of calcium absorption in the intestine. Currently, 11,031 potential VDR target genes have been identified [48]. Among these, 43% are associated with metabolic processes, 19% with cell and tissue morphology, 10% with cell junctions and adhesion, another 10% with differentiation and development, 9% with angiogenesis, and 5% with epithelial-to-mesenchymal transition. Furthermore, the VDR governs various microRNAs (miRNAs) and long non-coding RNAs linked to directly or indirectly expressing a broad spectrum of proteins. These insights collectively highlight VD’s vital role in numerous biological processes [47,49,50,51,52] (Figure 2).

VD plays a crucial role in calcium–phosphate homeostasis and bone mineralization while also demonstrating increasingly recognized functions in immune regulation, metabolic control, and cellular differentiation [53,54]. Its immunomodulatory effects include enhancing immune organ function and T-cell activity, thereby strengthening antitumor immunity through VDR expressed on immune cells. These receptors regulate genes involved in cell proliferation while suppressing tumor survival, migration, and metastasis [19]. In the context of hematopoietic stem cell transplantation (HSCT), VD deficiency, commonly observed both before and after transplantation, has been associated with increased risks of graft-versus-host disease (GVHD) and poorer survival outcomes. Conversely, maintaining sufficient VD levels during the transplantation process is correlated with reduced GVHD incidence, decreased production of pro-inflammatory cytokines, and enhanced immune reconstitution [55,56,57].

Regarding inflammation modulation, clinical evidence demonstrates that VD supplementation effectively attenuates oxidative stress and inflammatory markers (IL-6, hs-CRP, PAI-1, and fibrinogen) in VD-deficient patients with type 2 diabetes [58]. Furthermore, VD exhibits protective effects against cancer-associated inflammation, particularly in colorectal cancer [19], and provides cardiovascular benefits by reducing atherosclerotic risks, especially ischemic heart disease [59,60,61]. Notably, higher serum 25(OH)D levels show significant association with decreased venous thromboembolism risk, with enhanced protection observed in diabetic populations [49]. Additionally, VD contributes substantially to maternal health and fetal development [54,62].

Given these broad systemic functions, recent research has turned to exploring how VD status contributes to more complex physiological and pathological processes, particularly in the fields of cancer immunotherapy and host–microbiota interactions. The following sections will discuss these emerging domains of VD biology.

## 4. Effects of VD-Based Cancer Immunotherapy

Cancer remains a formidable global public health challenge in contemporary medicine [63,64]. Modern oncological interventions typically employ a multimodal approach, integrating surgical resection, chemotherapy, radiotherapy, hormone therapy, immunotherapy, and hematopoietic stem-cell transplantation to combat malignant progression. Immunotherapy is one of the oncological treatments that harnesses the immune system to selectively target tumor cells [24,25,26,65]. However, treatment responses are often heterogeneous. Considering its established roles in calcium–phosphate metabolism and systemic homeostasis, VD has emerged as a key immunomodulatory agent. In this context, VD has attracted attention for its potential to both enhance antitumor immunity and mitigate immunotherapy-associated toxicity [66].

The association between VD and cancer presents complex and varied findings. While multiple observational studies have found that breast cancer patients generally exhibit lower serum 25(OH)D levels compared with healthy controls, with more severe VD deficiency observed in advanced-stage patients [27,28,67], conclusions across studies differ significantly. Recent research has unveiled the population-specific nature of VD’s anticancer effects; a meta-analysis showed that higher serum 25(OH)D levels demonstrated significant protective effects only in premenopausal women or at the time of diagnosis [20]. Conversely, a cohort study focusing on elderly European populations reached the opposite conclusion, finding that breast cancer risk increased with higher 25(OH)D concentrations [68]. More strikingly, research by Kanstrup et al. indicated that female breast cancer patients with excessively high serum 25(OH)D levels (exceeding 110 nmol/L) exhibited poorer survival outcomes [8]. At the mechanistic level, most evidence supports VD’s role in inhibiting Th17 cell differentiation and IL-17 production via the VDR signaling pathway, thereby mitigating inflammatory responses and suppressing tumor progression. However, under certain conditions (such as in younger individuals or high-estrogen environments), VD may paradoxically promote Th17 cell activation and tumor metastasis risk by upregulating osteopontin (OPN) or activating estrogen receptor pathways, among other mechanisms [9]. Recent studies have revealed that VDR gene polymorphisms show significant associations with the risk of acute GVHD and patient survival following allogeneic bone marrow transplantation. Recipients carrying low-activity VDR alleles demonstrate increased susceptibility to severe GVHD. At the same time, donors with high-activity VDR genotypes significantly elevate recipient mortality risk, particularly in cases receiving intensified immunosuppressive therapy [69]. These conflicting findings suggest that VD’s role in cancer development and progression may be influenced by various factors, including age, hormone levels, and genetic background. VD’s precise mechanisms and clinical applications in oncology warrant further in-depth research.

Cancer immunotherapy represents a paradigm shift in oncology, fundamentally transforming cancer treatment strategies. This innovative approach harnesses and enhances the host’s immune system to target and eliminate malignant cells specifically, establishing itself as a cornerstone in contemporary cancer management [1]. Conventional immunotherapeutic strategies primarily focus on immune cell activation and immune response potentiation through several mechanisms, as follows: immune checkpoint blockade targeting CTLA-4 and PD-1/PD-L1 pathways to counteract immune evasion; tumor vaccines for immune system priming; adoptive cell therapy involving ex vivo immune cell modification and expansion; and monoclonal antibody-mediated specific antigen targetin [2,65,70,71,72]. The therapeutic advantages of immunotherapy are substantial, characterized by its exceptional specificity and minimal off-target effects on normal tissues. Clinically, this modality has demonstrated remarkable efficacy, with some patients achieving durable remission or complete eradication of malignancies [73]. Furthermore, the immunological memory conferred by this approach provides sustained protection against tumor recurrence, representing a significant advancement in cancer therapeutics [74].

VD demonstrates synergistic potential in cancer immunotherapy, primarily mediated through its interaction with the VDR. It is broadly expressed across immune cell lineages, including T lymphocytes, dendritic cells, and macrophages [75,76]. Studies reveal that the activation of the VDR signaling pathway enhances antitumor immune responses via dual mechanisms: it promotes the differentiation, maturation, and functional optimization of regulatory T cells (Tregs) while concurrently reducing immunosuppressive factor levels within the tumor microenvironment [64,77]. Notably, VD upregulates the expression of major histocompatibility complex (MHC) molecules, thereby significantly enhancing the immune system’s recognition capacity [78,79,80]. Additionally, the crosstalk regulatory network between VD and key signaling pathways such as PPARγ, PI3K/AKT/mTOR can dynamically modulate the expression of immune checkpoint molecules like PD-L1, providing new targets for combination therapies. Emerging evidence also reveals the synergistic tumor-suppressive effects between VD signaling and the estrogen receptor (ESR) pathway. These findings suggest that VD supplementation, through multi-pathway synergistic effects, may serve as an ideal adjuvant to enhance the efficacy of immune checkpoint blockade therapies [6].

**Figure 3:**
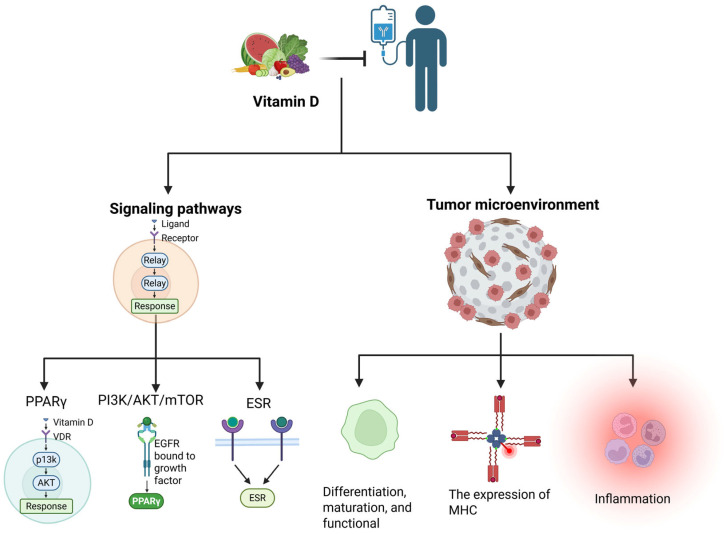
VD demonstrates potential in cancer immunotherapy. Its mechanisms of action include (1) interacting with pathways such as PPARγ/PI3K/ESR to regulate immune checkpoints like PD-L1; (2) modulating immune cell function; (3) upregulating MHC molecule expression to enhance immune recognition; and (4) suppressing pro-inflammatory factors such as IL-6/TNF-α. These multi-pathway synergistic effects suggest VD could serve as an ideal adjuvant to enhance the efficacy of immune checkpoint inhibitors.

The tumor microenvironment’s inflammatory milieu constitutes a pivotal driver of oncogenesis, facilitating neoplastic progression through sustained tumor cell proliferation, angiogenic induction, and metastatic dissemination [81]. In this pathological context, VD exerts multimodal anti-inflammatory effects, notably suppressing IL-6 and TNF-α production to disrupt the self-perpetuating cycle of inflammation-mediated tumorigenesis [29,82,83]. These immunomodulatory mechanisms collectively position VD as a potential adjuvant capable of recalibrating immune homeostasis to potentiate conventional immunotherapies (Figure 3).

In immunotherapy, particularly immune checkpoint inhibitors (ICIs) such as anti-PD-1/PD-L1 and anti-CTLA-4 therapies [4], studies have shown that maintaining VD levels within the normal range during anti-PD-1 immunotherapy is necessary to ensure treatment efficacy in patients with advanced melanoma [37,84]. Additional research has indicated that, for melanoma patients receiving PD-1, CTLA-4, or combined ICIs, the administration of VD significantly decreases the likelihood of developing ICI-induced colitis [4]. The PROVIDENCE study highlights that early implementation of systematic VD supplementation may exert beneficial effects on clinical outcomes in advanced cancer patients undergoing ICI therapy while also demonstrating that maintaining optimal VD sufficiency could serve as a preventive strategy against thyroid-associated immune-related adverse events (irAEs) [85]. Clinical observations have also suggested that higher serum levels of VD are associated with improved responses to immunotherapy and better overall survival in cancer patients [4,21,86].

However, the relationship between VD and cancer immunotherapy is complex and context-dependent. While VD demonstrates immunomodulatory benefits that may enhance immune checkpoint blockade therapy, emerging evidence suggests it may also exert immunosuppressive effects on dendritic cells (DCs) and β-cell function, which could potentially limit its therapeutic efficacy in certain contexts [8,87]. The current literature presents conflicting findings regarding VD’s role in cancer immunotherapy, with variations in study outcomes potentially attributable to differences in dosing regimens, patient characteristics [54], and cancer types [21]. Further research is needed to establish optimal dosing protocols, determine the most effective timing for VD administration relative to treatment cycles, elucidate its precise mechanisms of action, and identify patient subgroups that may derive the most significant clinical benefit from VD supplementation in combination with immunotherapy.

In conclusion, VD constitutes a promising adjunctive therapy in cancer immunotherapy, demonstrating the potential to bolster immune responses and enhance therapeutic efficacy. However, its complex and context-dependent effects require further clarification about its interplay with the gut microbiome, an emerging axis of immunoregulation in cancer.

## 5. VD Interacts with Gut Microbiota

Based on its role in modulating the immune system, VD also profoundly affects the gut microbiota. The interaction between VD and the gut microbiota may further illuminate VD’s contribution to cancer immunotherapy efficacy.

The gut microbiota, a complex and dynamic ecosystem residing within the host’s gastrointestinal tract, comprise various microorganisms, including bacteria, archaea, fungi, viruses, and bacteriophages. This intricate microbial community is pivotal in modulating multiple host physiological functions and is intimately involved in health maintenance and disease pathogenesis [88,89,90].

In the realm of nutritional metabolism, the gut microbiota play an indispensable role in nutrient biotransformation, particularly through the fermentation of indigestible dietary fibers into biologically active short-chain fatty acids (SCFAs) [91,92], while simultaneously orchestrating lipid metabolism and energy homeostasis. Regarding immune regulation, commensal microorganisms are essential for the maturation and differentiation of immune cells, particularly in gut-associated lymphoid tissues [36]. Furthermore, these microbial communities contribute to maintaining intestinal epithelial barrier integrity and regulating mucosal immune homeostasis through complex host–microbe interactions [93]. Meanwhile, they play significant roles in digesting food, regulating intestinal endocrine function and neural signaling [94], training host immunity [36], and modifying drug action and metabolism [95], as well as detoxifying the body [89]. However, although the gut microbiota play many key roles, they are complexly influenced by various physiological and environmental factors.

**Figure 4:**
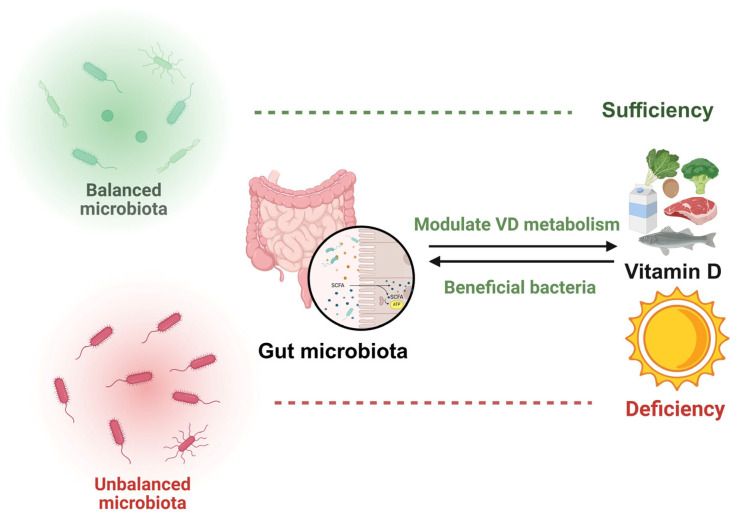
VD and gut microbiota exhibit a bidirectional regulatory relationship. VD can optimize gut microbiota composition, while the gut microbiota, in turn, regulates VD metabolic activity.

VD influences the gut microbiota apparently (Figure 4) [17,95]. The findings reveal a correlation between VD levels and the composition, diversity, or functionality of the gut microbiota [22,23,96]. In a double-blind randomized controlled trial, VD was found to improve gut microbiota and promote muscle anabolism [39]. The Mediterranean diet was applied to 91 patients with obesity and metabolic syndrome. After one year of dietary intervention, patients with low levels of 25(OH)D exhibited an increase in the diversity of their intestinal microbiota, which influenced their metabolic processes [30]. Additionally, individuals with the highest versus lowest concentrations of 1,25(OH)2D and its activation ratios tend to possess greater abundances of butyrate-producing bacteria, which have been linked to improved gut microbial health [40]. In particular, VD supplements can increase beneficial gut microbiota, including *Ruminococcaceae*, *Akkermansia*, *Faecalibacterium*, and *Coccus*, thereby modulating autoimmune responses [41]. In mice subjected to VD-deficient diets or genetic knockout models, the abundance of *Bacteroidetes* (or taxa within this phylum) in the gut microbiota was observed to increase [23]. VD induces the expression of antimicrobial peptides (AMPs) in the zebrafish intestine by activating microbiota-dependent IL-22 signaling. In VD-deficient zebrafish, the abundance of the acetate-producing bacterium *Vibrio* is reduced. This study demonstrates that VD regulates the composition of the gut microbiota in zebrafish and the production of short-chain fatty acids (SCFAs), thereby enhancing immunity [92]. VD deficiency syndrome can manifest as colonic hyperplasia and epithelial barrier dysfunction [97].

VDR is also closely related to the gut microbiota [17]. The downregulation of VDR and the impaired ability to produce the active form of VD have been correlated with a reduction in *Lactobacillus* and an increase in *Proteobacteria* within the gut microbiota [42]. In addition, other research indicates that the induction of Cyp27b1 in mice colonic epithelial cells, which is expected to boost local production of 1,25(OH)2D, functions as a protective mechanism. This mechanism partially mitigates the downregulation of epithelial VDR during colonic inflammation. Elevated local levels of 1,25(OH)2D sustain the 1,25(OH)2D-VDR signaling pathway, which safeguards the mucosal barrier and diminishes colonic inflammation [43]. In parallel, fecal and cecal stool samples were collected from VDR knockout (Vdr^−/−^) and wild-type mice to extract bacterial DNA. Then, samples were subjected to 454 pyrosequencing to determine the bacterial composition present in the stool samples. The findings suggest that VDR status influences the gut microbiota at a taxonomic and functional levels and correlates with VDR-associated bacterial changes in clinical disease [98].

Studies have shown that gut microbiota can modulate intestinal VD metabolism [18]. Specifically, the *Bifidobacterium longum* strain FSHHK13M significantly elevated 1,25-dihydroxy VD and osteocalcin serum, thereby alleviating osteoporosis in mice [99]. In addition, the study found that doubling the genetic liability associated with the abundance of *Erysipelotrichia*, *Erysipelotrichaceae*, and *Erysipelotrichales* reduced the concentration of 25(OH)D [100].

The literature, thus, suggests a potential pathogenic cascade: VD deficiency triggers gut microbiota imbalance, exacerbating microbial dysbiosis and systemic disease [96]. Furthermore, the gut microbiota reciprocally modulate intestinal VD levels. This bidirectional interplay between VD and gut microbiota may inform clinical strategies to optimize VD supplementation.

Given the central role of the gut microbiota in immune modulation and their tight interplay with VD signaling, it is reasonable to hypothesize that VD–microbiota interactions may significantly influence responses to cancer immunotherapy. The following section explores how gut microbiota composition and function affect the efficacy and safety of cancer immunotherapy, thereby complementing the immunoregulatory role of VD.

## 6. Gut Microbiota as a Determinant of Immunotherapy Efficacy

**Figure 5:**
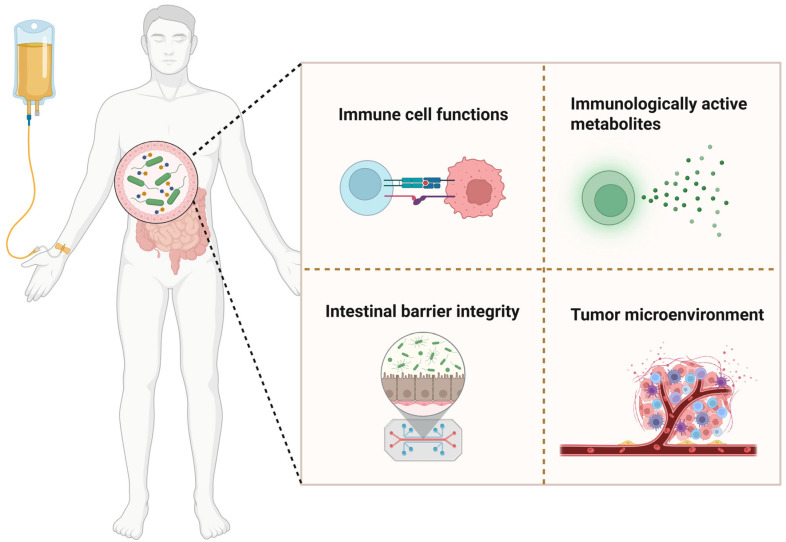
Gut microbiota impact on cancer immunotherapy. The gut microbiota serve as a critical regulator of cancer immunotherapy efficacy through the following mechanisms: (1) regulating immune cell functions; (2) generating immunologically active metabolites; (3) preserving intestinal barrier integrity; (4) reshaping the tumor microenvironment.

The gut microbiota have emerged as a key modulator of cancer immunotherapy efficacy. Research has demonstrated that gut microbiota profoundly influence the effectiveness of immunotherapy through multiple mechanisms [10], including modulation of immune cell function, production of immunologically active metabolites, maintenance of intestinal barrier integrity, and alteration of the tumor microenvironment (Figure 5). Firstly, concerning immune cell function, studies have demonstrated that secondary bile acids, which are products of primary bile acid metabolism by gut microbiota, enhance the activation and effector functions of T cells while reducing the accumulation and functionality of myeloid-derived suppressor cells (MDSCs) [101,102], thereby modulating immune cell activity. In terms of the production of immunologically active metabolites, gut microbiota such as *Akkermansia muciniphila* and *Bifidobacterium* enhance the antitumor activity of CD8+ T cells by producing short-chain fatty acids (SCFAs) and tryptophan metabolites, thus improving the efficacy of immunotherapy [15,16]. Analysis of fecal metagenomes from 112 melanoma patients and in vivo experiments in mice revealed that bacterial subpopulations encoding immunostimulatory hexa-acylated lipopolysaccharide (LPS) can enhance the antitumor efficacy of anti-PD-1 therapy [31]. Additionally, the gut microbiota support intestinal barrier integrity, blocking bacterial and toxin entry into the bloodstream to reduce systemic inflammation and indirectly enhance antitumor immunity [32]. Finally, research has shown that the gut microbiota can alter the tumor microenvironment; for instance, *Bacteroides fragilis* enhances the efficacy of anti-CTLA-4 therapy by remodeling the tumor microenvironment through immunomodulatory mechanisms [11].

Many findings highlight the critical role of gut microbiota in modulating host immune responses and shaping clinical outcomes during cancer immunotherapy [12,13]. Emerging evidence positions the gut microbiota as a vital determinant in regulating therapeutic responses to ICIs [15,16,33]. Fecal microbiota transplantation (FMT) from humans to mice demonstrated that anti-CTLA-4 antibody treatment in melanoma patients promotes the expansion of *Bacteroides fragilis*, which possesses potent anticancer properties [11]. In previous studies, extensive research has shown that the gut microbiota composition serves as a reliable predictive biomarker for both the therapeutic efficacy of immune checkpoint blockade therapy and the likelihood of associated adverse events.

In summary, the gut microbiota play a critical role in cancer immunotherapy. Looking ahead, modulating the gut microbiota through approaches such as probiotics and FMT may emerge as a pivotal strategy for enhancing therapeutic efficacy and reducing adverse effects in immunotherapy.

Importantly, VD is a potential co-regulator of this microbiota–immune axis to enhance responses in cancer immunotherapy. The following section thoroughly explores this emerging interplay between VD and gut microbiota.

## 7. Gut Microbiota and VD Synergy in Modulating Cancer Immunotherapy

Recent studies have underscored the pivotal role of VD in immune regulation and its profound implications for cancer immunity [34,103,104]. Research by Giampazolias et al. [7] demonstrates that elevated VD levels enhance immune-mediated resistance to melanoma and improve responses to immune checkpoint blockade therapies. This immunomodulatory effect is mediated through VD’s action on intestinal epithelial cells (IECs), which remodel the gut microbiota and promote the proliferation of *Bacteroides fragilis*, a bacterium known for its positive regulation of cancer immunity.

Animal studies reveal that VD deficiency or supplementation directly impacts gut microbiota composition, triggering significant immune response alterations. VD facilitates the expansion of *Bacteroides fragilis* and reduces the abundance of *Prevotella brevis*. This microbial shift markedly enhances antitumor immunity by boosting T-cell activity and improving immunotherapy efficacy. These beneficial effects can be transferred via FMT, provided recipients maintain adequate dietary VD levels. This indicates that VD establishes a conducive microenvironment in the gut that supports the growth of beneficial bacteria like *Bacteroides fragilis*, thereby amplifying cancer immune responses. In human studies, VD levels and the expression of VD receptor (VDR) target genes correlate with improved cancer prognoses and enhanced efficacy of ICIs [7,103,104].

**Figure 6:**
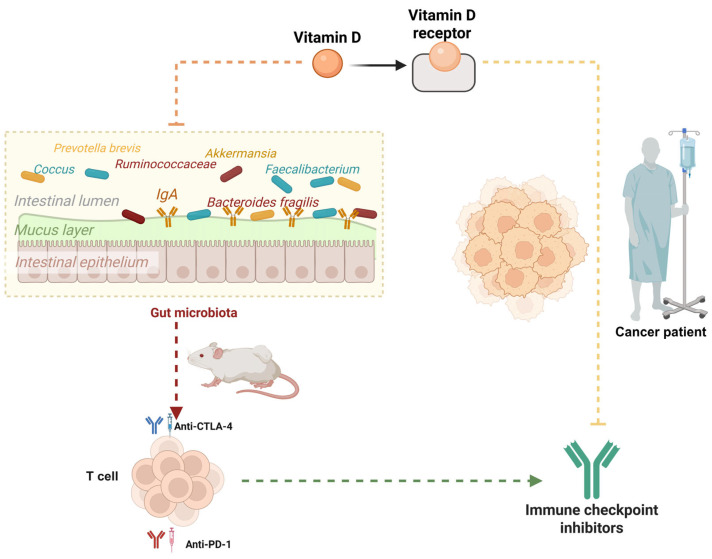
VD-dependent microbiota-enhancing tumor immunotherapy. VD levels act on intestinal epithelial cells to promote the growth of beneficial bacteria (e.g., *Bacteroides fragilis*) while inhibiting the proliferation of unfavorable bacterial species (e.g., *Prevotella brevis*). This microbiota remodeling effect significantly enhances T-cell activity and improves the efficacy of immunotherapy responses. Clinical evidence demonstrates that VD status and VDR target gene expression correlate with improved therapeutic outcomes in patients.

In summary, these findings suggest that VD status may serve as a predictive biomarker for immunotherapy outcomes and a potential therapeutic target to optimize cancer treatment [35,37]. This highlights the strategic potential of VD supplementation in modulating the microbiota and augmenting the effectiveness of cancer immunotherapies (Figure 6). Further clinical translational research is warranted to explore optimal approaches for VD supplementation in cancer patients.

## 8. Publication Bias and Evidence Gaps

Publication bias represents a critical yet underrecognized challenge in studies investigating the role of VD in cancer immunotherapy. The current scientific literature exhibits a marked overrepresentation of positive findings demonstrating VD’s immunomodulatory or therapeutic benefits compared with neutral or negative results [4,8,9]. This systematic imbalance not only risks inflating perceptions of VD’s clinical efficacy but may also obscure essential limitations and paradoxical effects. While numerous observational studies report positive correlations between VD status and immunotherapy response, research documenting null effects or potential adverse outcomes, such as immunosuppression at high VD doses [87] or negative survival associations [8], frequently receives inadequate attention or remains unpublished.

Several methodological factors contribute to this imbalance. Statistically significant positive findings predominantly emerge from small-scale or non-randomized controlled trials [21], whereas large-scale randomized controlled trials (RCTs) yielding neutral outcomes often encounter publication barriers. Additionally, industry-sponsored studies may demonstrate preferential reporting of favorable results [84]. Addressing these evidence gaps requires a concerted research effort focusing on the following three key areas: implementing rigorous null-result trial designs with prospective registration and publication protocols [47]; conducting multicenter, large-scale RCTs to validate VD’s dose–response relationships, population-specific effects [85], and gut microbiota interactions [7]; and establishing standardized evaluation metrics for both VD status assessment and immunotherapy response measurement to minimize interpretation bias arising from methodological heterogeneity [47].

This comprehensive approach will enable more accurate characterization of VD’s therapeutic potential in cancer immunotherapy while mitigating current biases in the evidence base. Such efforts are particularly crucial given the growing clinical interest in VD as an immunomodulatory adjuvant, where balanced evaluation of both benefits and risks is essential for informed treatment decisions [85]. Future research must prioritize methodological rigor and transparency to overcome existing publication biases and establish a more reliable evidence foundation for clinical applications.

## 9. Discussion and Conclusions

The synergistic interplay among VD, gut microbiota, and cancer immunotherapy represents a promising frontier in tumor treatment research [7]. VD exerts its effects through the following dual mechanisms: directly modulating immune cell activity via the VDR and reshaping gut microbiota composition to promote colonization of beneficial species such as *Bacteroides fragilis* [99]. This “microbiota-immune axis” enhancement augments antitumor immune responses, with optimal VD levels having been shown to improve the efficacy of ICIs, reduce immune-related toxicities, and prolong patient survival [37,85].

However, VD’s role in immunotherapy exhibits notable paradoxes. While it typically suppresses the Th17/IL-17 inflammatory pathway to hinder tumor progression [19], context-dependent effects such as enhanced Th17 activity and increased metastasis risk in specific populations or tumor types highlight the complexity of its immunomodulatory profile [9].

These contradictory effects are rooted in multiple interdependent factors, starting with host-related influences, where younger patients demonstrate heightened sensitivity to VD-mediated immune regulation compared with older individuals with blunted responses due to immunosenescence [71]; for example, high-estrogen environments in younger patients may prime VD to activate Th17 pathways and paradoxically promote tumor metastasis [9]. Gender and hormonal milieu also play a role, as estrogen levels influence VD’s immunomodulatory trajectory (evident in breast cancer through crosstalk between estrogen receptor and VDR signaling) [69] and sex-based disparities in VD metabolism impact therapeutic efficacy.

Therapeutic- and tumor-related factors further complicate this complexity. VD exhibits a biphasic dose–response, where low doses enhance antitumor immunity, while high doses (serum 25(OH)D > 110 nmol/L) in breast cancer correlate with immunosuppression [8]. Tumor heterogeneity leads to divergent responses, such that VD enhances immunotherapy in melanoma and colorectal cancer via modulation of the gut microbiota but exhibits paradoxical effects in hormone-dependent cancers [15,68].

Microbial and genetic determinants are equally critical. VD promotes growth of beneficial bacteria like butyrate-producing species while microbiota metabolize VD into active 1,25(OH)_2_D in a bidirectional regulatory loop [40], with interindividual microbiota variations underlying inconsistent VD efficacy [13]; meanwhile, VDR gene polymorphisms alter VD signaling efficiency, contributing to interpatient differences in cancer risk and immunotherapy response [69].

In summary, VD’s dual role in cancer immunotherapy is governed by a complex matrix of host, tumor, microbial, and genetic factors [7], the deciphering of which may enable personalized VD supplementation strategies to optimize immunotherapeutic outcomes while mitigating paradoxical effects.

## 10. Future Perspectives

Although the synergy between VD and immunotherapy shows great promise, several key issues remain to be addressed, as follows:

(a) Mechanistic Research: Further exploration is needed to understand how VD influences immune cell function via gut microbiota, particularly its bidirectional regulation of the Th17/Treg balance [9,86]. Studies should also investigate VD’s crosstalk with other critical signaling pathways (e.g., PPARγ and PI3K/AKT/mTOR) and its dynamic impact on PD-L1 expression [6,35].
(b) Personalized Treatment Strategies: Multi-omics data (e.g., metagenomics, metabolomics, and immunomics) should be integrated to develop predictive models identifying patients who may benefit from VD supplementation [13,15]. Precision intervention strategies, such as combining probiotics, prebiotics, or FMT, should be explored to optimize immunotherapy outcomes [16,33].
(c) Clinical Translation: Large-scale RCTs are required to determine the optimal VD dosage, timing, and target populations while avoiding the immunosuppressive risks of excessive supplementation [34,85]. The combined use of VD with other immunomodulators should be investigated to develop more effective combination therapies [6].
(d) Technological Advancements: Rapid and cost-effective VD and gut microbiota detection methods should be developed to facilitate routine clinical monitoring [47]. Organoid or humanized mouse models could help simulate VD–microbiota–immune system interactions, accelerating mechanistic research.

In summary, integrating VD and immunotherapy provides a promising new approach to cancer treatment, but its clinical application requires deeper mechanistic insights and rigorous validation. Combining basic research, multi-omics analysis, and personalized medicine, safer and more effective precision immunotherapy strategies may soon become a reality.

## Figures and Tables

**Table 1:** Quality assessment of AMSTAR.

Author/Year	①	②	③	④	⑤	⑥	⑦	⑧	⑨	⑩	⑪	Total Score	Literature Quality
Fekete M. et al., 2025 [19]	1	0	1	1	1	1	0	0	0	0	1	6	Medium
Estébanez N. et al., 2018 [20]	1	1	1	1	1	1	1	1	1	1	1	11	High
Mondul A.M. et al., 2017 [21]	0	0	1	0	1	1	0	0	1	1	1	6	Medium
Aggeletopoulou I. et al., 2023 [22]	1	1	1	1	0	1	0	1	1	0	1	8	High
Waterhouse M. et al., 2018 [23]	1	1	1	1	1	1	1	1	1	0	1	10	High

① Is there a study design protocol? ② Were screening and extraction performed by two individuals? ③ Is the search strategy comprehensive? ④ Do the inclusion criteria cover gray literature? ⑤ Are there inclusion and exclusion criteria? ⑥ Are the characteristics of the included studies described? ⑦ Were the scientific quality of the included studies assessed and reported? ⑧ Was the scientific quality of the included studies appropriately applied in deriving conclusions? ⑨ Was the synthesis of the results appropriate? ⑩ Was the possibility of publication bias assessed? ⑪ Were conflicts of interest disclosed?

**Table 2:** Quality assessment of NOS.

Author/Year	Selection	Comparability	Outcome	Total Score	Literature Quality
Grover S. et al., 2020 [4]	★★★★	★★	★★★	Nine	High
Kanstrup C. et al., 2020 [8]	★★★★	★★	★★★	Nine	High
Chaput N. et al., 2017 [12]	★★★	★	★★★	Seven	High
Zhu X. et al., 2024 [13]	★★★★	★★	★★★	Nine	High
Gopalakrishnan V. et al., 2018 [15]	★★★★	★★	★★★	Nine	High
Matson V. et al., 2018 [16]	★★★★	★★	★★★	Nine	High
Song S. et al., 2025 [24]	★★★★	★★	★★★	Nine	High
Lim S.T. et al., 2015 [25]	★★★	★★	★	Six	Medium
Shirazi L. et al., 2016 [26]	★★★	★★	★★★	Eight	High
Ordóñez-Mena J.M. et al., 2016 [27]	★★★★	★★	★★★	Nine	High
Middleton P.G. et al., 2002 [28]	★★★★	★★	★★★	Nine	High
Galus Ł. et al., 2023 [29]	★★★★	★★	★★	Eight	High
Boughanem H. et al., 2023 [30]	★★★	★★	★★★	Eight	High
Sardar P. et al., 2025 [31]	★★★★	★★	★★	Eight	High
Zitvogel L. et al., 2018 [32]	★★★	★★	★★	Seven	High
Routy B. et al., 2018 [33]	★★★★	★★	★★	Eight	High
Kanno K. et al., 2023 [34]	★★★★	★★	★★★	Nine	High
Ferrer-Mayorga G. et al., 2017 [35]	★★★★	★★	★★	Eight	High

**Table 3:** Quality assessment of modified Jadad score.

Author/Year	Random Sequence Generation	Randomization Concealment	Blinding	Withdrawals and Dropouts	Total Score	Literature Quality
Jamshidi S. et al., 2022 [36]	+2	+2	+2	+1	7	High

**Table 4:** Quality assessment of SYECLE.

Author/Year	Sequence Generation	Baseline Characteristics	Allocation Concealment	Random Housing	Blinding	Random Outcome Assessment	Incomplete Outcome Data	Selective Outcome Reporting	Other Bias
Giampazolias E. et al., 2024 [7]	High	Low	Unclear	Unclear	Unclear	Low	Low	Low	Moderate
Vétizou M. et al., 2015 [11]	Unclear	Low	Unclear	Low	Unclear	Low	Low	Low	Low
Gopalakrishnan V. et al., 2018 [15]	Unclear	Low	Unclear	Low	Unclear	Low	Low	Low	Low
Matson V. et al., 2018 [16]	Unclear	Low	Unclear	Low	Unclear	Low	Low	Low	Low
Liao X. et al., 2023 [37]	Unclear	Unclear	Unclear	Low	Unclear	Low	Low	Low	Unclear
Assa A. et al., 2014 [38]	Unclear	Low	Unclear	Low	Unclear	Low	Low	Low	Unclear
Du J. et al., 2022 [39]	Unclear	Unclear	Unclear	Low	Unclear	Low	Low	Low	Low
Jin D. et al., 2015 [40]	Unclear	Unclear	Unclear	Low	Unclear	Low	Low	Low	Low
Wang H. et al., 2025 [41]	Unclear	Low	Unclear	Low	Unclear	Low	Low	Low	Unclear
Ma C. et al., 2018 [42]	Unclear	Low	Unclear	Low	Unclear	Low	Low	Low	Low
Song X. et al., 2020 [43]	Unclear	Low	Unclear	Low	Unclear	Low	Low	Low	Low

## Data Availability

The data presented in this study are available upon request from the corresponding author.

## References

[1] Kennedy L.B., Salama A.K.S. (2020). A review of cancer immunotherapy toxicity. CA Cancer J. Clin..

[2] Szeto G.L., Finley S.D. (2019). Integrative Approaches to Cancer Immunotherapy. Trends Cancer.

[3] Bouillon R., Marcocci C., Carmeliet G., Bikle D., White J.H., Dawson-Hughes B., Lips P., Munns C.F., Lazaretti-Castro M., Giustina A. (2019). Skeletal and Extraskeletal Actions of Vitamin D: Current Evidence and Outstanding Questions. Endocr. Rev..

[4] Grover S., Dougan M., Tyan K., Giobbie-Hurder A., Blum S.M., Ishizuka J., Qazi T., Elias R., Vora K.B., Ruan A.B. (2020). Vitamin D intake is associated with decreased risk of immune checkpoint inhibitor-induced colitis. Cancer.

[5] Ghaseminejad-Raeini A., Ghaderi A., Sharafi A., Nematollahi-Sani B., Moossavi M., Derakhshani A., Sarab G.A. (2023). Immunomodulatory actions of vitamin D in various immune-related disorders: A comprehensive review. Front. Immunol..

[6] Tsuji A., Yoshikawa S., Morikawa S., Ikeda Y., Taniguchi K., Sawamura H., Asai T., Matsuda S. (2023). Potential tactics with vitamin D and certain phytochemicals for enhancing the effectiveness of immune-checkpoint blockade therapies. Explor. Target. Antitumor Ther..

[7] Giampazolias E., Pereira da Costa M., Lam K.C., Lim K.H.J., Cardoso A., Piot C., Chakravarty P., Blasche S., Patel S., Biram A. (2024). Vitamin D regulates microbiome-dependent cancer immunity. Science.

[8] Kanstrup C., Teilum D., Rejnmark L., Bigaard J.V., Eiken P., Kroman N., Tjønneland A., Mejdahl M.K. (2020). 25-Hydroxyvitamin D at time of breast cancer diagnosis and breast cancer survival. Breast Cancer Res. Treat..

[9] Filip-Psurska B., Zachary H., Strzykalska A., Wietrzyk J. (2022). Vitamin D, Th17 Lymphocytes, and Breast Cancer. Cancers.

[10] Fessler J., Matson V., Gajewski T.F. (2019). Exploring the emerging role of the microbiome in cancer immunotherapy. J. Immunother. Cancer.

[11] Vétizou M., Pitt J.M., Daillère R., Lepage P., Waldschmitt N., Flament C., Rusakiewicz S., Routy B., Roberti M.P., Duong C.P. (2015). Anticancer immunotherapy by CTLA-4 blockade relies on the gut microbiota. Science.

[12] Chaput N., Lepage P., Coutzac C., Soularue E., Le Roux K., Monot C., Boselli L., Routier E., Cassard L., Collins M. (2017). Baseline gut microbiota predicts clinical response and colitis in metastatic melanoma patients treated with ipilimumab. Ann. Oncol..

[13] Zhu X., Huang X., Hu M., Sun R., Li J., Wang H., Pan X., Ma Y., Ning L., Tong T. (2024). A specific enterotype derived from gut microbiome of older individuals enables favorable responses to immune checkpoint blockade therapy. Cell Host Microbe.

[14] Park E.M., Chelvanambi M., Bhutiani N., Kroemer G., Zitvogel L., Wargo J.A. (2022). Targeting the gut and tumor microbiota in cancer. Nat. Med..

[15] Gopalakrishnan V., Spencer C.N., Nezi L., Reuben A., Andrews M.C., Karpinets T.V., Prieto P.A., Vicente D., Hoffman K., Wei S.C. (2018). Gut microbiome modulates response to anti–PD-1 immunotherapy in melanoma patients. Science.

[16] Matson V., Fessler J., Bao R., Chongsuwat T., Zha Y., Alegre M.L., Luke J.J., Gajewski T.F. (2018). The commensal microbiome is associated with anti–PD-1 efficacy in metastatic melanoma patients. Science.

[17] Ullah H. (2025). Gut-vitamin D interplay: Key to mitigating immunosenescence and promoting healthy ageing. Immun. Ageing.

[18] Barbáchano A., Fernández-Barral A., Ferrer-Mayorga G., Costales-Carrera A., Larriba M.J., Muñoz A. (2017). The endocrine vitamin D system in the gut. Mol. Cell Endocrinol..

[19] Fekete M., Lehoczki A., Szappanos Á., Zábó V., Kaposvári C., Horváth A., Farkas Á., Fazekas-Pongor V., Major D., Lipécz Á. (2025). Vitamin D and Colorectal Cancer Prevention: Immunological Mechanisms, Inflammatory Pathways, and Nutritional Implications. Nutrients.

[20] Estébanez N., Gómez-Acebo I., Palazuelos C., Llorca J., Dierssen-Sotos T. (2018). Vitamin D exposure and Risk of Breast Cancer: A meta-analysis. Sci. Rep..

[21] Mondul A.M., Weinstein S.J., Layne T.M., Albanes D. (2017). Vitamin D and Cancer Risk and Mortality: State of the Science, Gaps, and Challenges. Epidemiol. Rev..

[22] Aggeletopoulou I., Tsounis E.P., Mouzaki A., Triantos C. (2023). Exploring the Role of Vitamin D and the Vitamin D Receptor in the Composition of the Gut Microbiota. Front. Biosci. (Landmark Ed.).

[23] Waterhouse M., Hope B., Krause L., Morrison M., Protani M.M., Zakrzewski M., Neale R.E. (2019). Vitamin D and the gut microbiome: A systematic review of in vivo studies. Eur. J. Nutr..

[24] Riley R.S., June C.H., Langer R., Mitchell M.J. (2019). Delivery technologies for cancer immunotherapy. Nat. Rev. Drug Discovery..

[25] Vanhooren J., Derpoorter C., Depreter B., Deneweth L., Philippé J., De Moerloose B., Lammens T. (2021). TARP as antigen in cancer immunotherapy. Cancer Immunol. Immunother..

[26] Paleari L. (2024). Personalized Assessment for Cancer Prevention, Detection, and Treatment. Int. J. Mol. Sci..

[27] Lim S.T., Jeon Y.W., Suh Y.J. (2015). Association Between Alterations in the Serum 25-Hydroxyvitamin D Status During Follow-Up and Breast Cancer Patient Prognosis. Asian Pac. J. Cancer Prev..

[28] Shirazi L., Almquist M., Borgquist S., Malm J., Manjer J. (2016). Serum vitamin D (25OHD3) levels and the risk of different subtypes of breast cancer: A nested case–control study. Breast.

[29] Kim D.H., Meza C.A., Clarke H., Kim J.S., Hickner R.C. (2020). Vitamin D and Endothelial Function. Nutrients.

[30] Boughanem H., Ruiz-Limón P., Pilo J., Lisbona-Montañez J.M., Tinahones F.J., Moreno Indias I., Macías-González M. (2023). Linking serum vitamin D levels with gut microbiota after 1-year lifestyle intervention with Mediterranean diet in patients with obesity and metabolic syndrome: A nested cross-sectional and prospective study. Gut Microbes.

[31] Sardar P., Beresford-Jones B.S., Xia W., Shabana O., Suyama S., Ramos R.J.F., Soderholm A.T., Tourlomousis P., Kuo P., Evans A.C. (2025). Gut microbiota-derived hexa-acylated lipopolysaccharides enhance cancer immunotherapy responses. Nat. Microbiol..

[32] Zitvogel L., Ma Y., Raoult D., Kroemer G., Gajewski T.F. (2018). The microbiome in cancer immunotherapy: Diagnostic tools and therapeutic strategies. Science.

[33] Routy B., Le Chatelier E., Derosa L., Duong C.P.M., Alou M.T., Daillère R., Fluckiger A., Messaoudene M., Rauber C., Roberti M.P. (2018). Gut microbiome influences efficacy of PD-1-based immunotherapy against epithelial tumors. Science.

[34] Kanno K., Akutsu T., Ohdaira H., Suzuki Y., Urashima M. (2023). Effect of Vitamin D Supplements on Relapse or Death in a p53-Immunoreactive Subgroup With Digestive Tract Cancer: Post Hoc Analysis of the AMATERASU Randomized Clinical Trial. JAMA Netw. Open.

[35] Fernández-Barral A., Peña C., Pisano D.G., Cantero R., Rojo F., Muñoz A., Larriba M.J. (2017). Vitamin D receptor expression and associated gene signature in tumour stromal fibroblasts predict clinical outcome in colorectal cancer. Gut.

[36] Fan L., Xia Y., Wang Y., Han D., Liu Y., Li J., Fu J., Wang L., Gan Z., Liu B. (2023). Gut microbiota bridges dietary nutrients and host immunity. Sci. China Life Sci..

[37] Galus Ł., Michalak M., Lorenz M., Stoińska-Swiniarek R., Tusień Małecka D., Galus A., Kolenda T., Leporowska E., Mackiewicz J. (2023). Vitamin D supplementation increases objective response rate and prolongs progression-free time in patients with advanced melanoma undergoing anti-PD-1 therapy. Cancer.

[38] Huang L., Lum D., Haiyum M., Fairbairn K.A. (2021). Vitamin D Status of Elite Athletes in Singapore and Its Associations With Muscle Function and Bone Health. J. Sci. Sport Exerc..

[39] Jamshidi S., Masoumi S.J., Abiri B., Vafa M. (2022). The effects of synbiotic and/or vitamin D supplementation on gut-muscle axis in overweight and obese women: A study protocol for a double-blind, randomized, placebo-controlled trial. Trials.

[40] Thomas R.L., Jiang L., Adams J.S., Xu Z.Z., Shen J., Janssen S., Ackermann G., Vanderschueren D., Pauwels S., Knight R. (2020). Vitamin D metabolites and the gut microbiome in older men. Nat. Commun..

[41] Clark A., Mach N. (2016). Role of Vitamin D in the Hygiene Hypothesis: The Interplay between Vitamin D, Vitamin D Receptors, Gut Microbiota, and Immune Response. Front. Immunol..

[42] Battistini C., Ballan R., Herkenhoff M.E., Saad S.M.I., Sun J. (2020). Vitamin D Modulates Intestinal Microbiota in Inflammatory Bowel Diseases. Int. J. Mol. Sci..

[43] Du J., Wei X., Ge X., Chen Y., Li Y.C. (2017). Microbiota-Dependent Induction of Colonic Cyp27b1 Is Associated With Colonic Inflammation: Implications of Locally Produced 1,25-Dihydroxyvitamin D3 in Inflammatory Regulation in the Colon. Endocrinology.

[44] Holick M.F., Chen T.C. (2008). Vitamin D deficiency: A worldwide problem with health consequences. Am. J. Clin. Nutr..

[45] Palacios C., Gonzalez L. (2014). Is vitamin D deficiency a major global public health problem?. J. Steroid Biochem. Mol. Biol..

[46] Roth D.E., Abrams S.A., Aloia J., Bergeron G., Bourassa M.W., Brown K.H., Calvo M.S., Cashman K.D., Combs G., De-Regil L.M. (2018). Global prevalence and disease burden of vitamin D deficiency: A roadmap for action in low- and middle-income countries. Ann. N. Y. Acad. Sci..

[47] Giustina A., Bilezikian J.P., Adler R.A., Banfi G., Bikle D.D., Binkley N.C., Bollerslev J., Bouillon R., Brandi M.L., Casanueva F.F. (2024). Consensus Statement on Vitamin D Status Assessment and Supplementation: Whys, Whens, and Hows. Endocr. Rev..

[48] Saccone D., Asani F., Bornman L. (2015). Regulation of the vitamin D receptor gene by environment, genetics and epigenetics. Gene.

[49] Xiang H., Zhou C., Gan X., Huang Y., He P., Ye Z., Liu M., Yang S., Zhang Y., Zhang Y. (2025). Relationship of Serum 25-Hydroxyvitamin D Concentrations, Diabetes, Vitamin D Receptor Gene Polymorphisms and Incident Venous Thromboembolism. Diabetes Metab. Res. Rev..

[50] Carlberg C., Raczyk M., Zawrotna N. (2023). Vitamin D: A master example of nutrigenomics. Redox Biol..

[51] Delrue C., Speeckaert M.M. (2023). Vitamin D and Vitamin D-Binding Protein in Health and Disease. Int. J. Mol. Sci..

[52] Thouvenot E., Laplaud D., Lebrun-Frenay C., Derache N., Le Page E., Maillart E., Froment-Tilikete C., Castelnovo G., Casez O., Coustans M. (2025). High-Dose Vitamin Din Clinically Isolated Syndrome Typical of Multiple Sclerosis: The D-Lay MSRandomized Clinical Trial. JAMA.

[53] Charoenngam N., Shirvani A., Holick M.F. (2019). Vitamin D for skeletal and non-skeletal health: What we should know. J. Clin. Orthop. Trauma.

[54] Demay M.B., Pittas A.G., Bikle D.D., Diab D.L., Kiely M.E., Lazaretti-Castro M., Lips P., Mitchell D.M., Murad M.H., Powers S. (2024). Vitamin D for the Prevention of Disease: An Endocrine Society Clinical Practice Guideline. J. Clin. Endocrinol. Metab..

[55] Olsen B., Bodea J., Garcia A., Beebe K., Campbell C., Schwalbach C., Salzberg D., Miller H., Adams R., Mirea L. (2022). Vitamin D Supplementation: Association With Serum Cytokines in Pediatric Hematopoietic Stem Cell Transplantation. Front. Pediatr..

[56] Ros-Soto J., Anthias C., Madrigal A., Snowden J.A. (2019). Vitamin D: Is it important in haematopoietic stem cell transplantation? A review. Bone Marrow Transplant..

[57] Bodea J., Beebe K., Campbell C., Salzberg D., Schwalbach C., Miller H., Adams R., Mirea L., Castillo P., Horn B. (2022). Impact of Adequate Day 30 Post-Pediatric Hematopoietic Stem Cell Transplantation Vitamin D Level on Clinical Outcome: An Observational Cohort Study. Transplant. Cell Ther..

[58] Pasupuleti P., Suchitra M.M., Bitla A.R., Sachan A. (2021). Attenuation of Oxidative Stress, Interleukin-6, High-Sensitivity C-Reactive Protein, Plasminogen Activator Inhibitor-1, and Fibrinogen with Oral Vitamin D Supplementation in Patients with T2DM having Vitamin D Deficiency. J. Lab. Physicians.

[59] Lin K., Miao Y., Gan L., Zhao B., Fang F., Wang R., Chen X., Huang J. (2025). Associations of Serum 25-Hydroxyvitamin D Concentrations with Risks of Mortality and Cardiovascular Disease among Individuals with Psoriasis. J. Am. Acad. Dermatol..

[60] Sha S., Xie R., Gwenzi T., Wang Y., Brenner H., Schöttker B. (2025). Real-world evidence for an association of vitamin D supplementation with atherosclerotic cardiovascular disease in the UK Biobank. Clin. Nutr..

[61] Ruiz-García A., Pallarés-Carratalá V., Turégano-Yedro M., Torres F., Sapena V., Martin-Gorgojo A., Martin-Moreno J.M. (2023). Vitamin D Supplementation and Its Impact on Mortality and Cardiovascular Outcomes: Systematic Review and Meta-Analysis of 80 Randomized Clinical Trials. Nutrients.

[62] Wang X., Li Q., Lyu Z., Wu Y. (2025). Supplementing with Vitamin D during Pregnancy Reduces Inflammation and Prevents Autism-Related Behaviors in Offspring Caused by Maternal Immune Activation. Biol. Pharm. Bull..

[63] Mullard A. (2020). Addressing cancer’s grand challenges. Nat. Rev. Drug Discov..

[64] Elkin E.B., Bach P.B. (2010). Cancer’s next frontier: Addressing high and increasing costs. JAMA.

[65] Yu Q., Ding J., Li S., Li Y. (2024). Autophagy in cancer immunotherapy: Perspective on immune evasion and cell death interactions. Cancer Lett..

[66] Song S., Woo H.D., Lyu J., Song B.M., Lim J.Y., Park H.Y. (2025). Serum 25-hydroxyvitamin D levels and risk of overall and site-specific cancers in Korean adults: Results from two prospective cohort studies. Nutr. J..

[67] Li H., Liu H., Wang B., Jia X., Yu J., Zhang Y., Sang D., Zhang Y. (2024). Exercise Interventions for the Prevention and Treatment of Anthracycline-Induced Cardiotoxicity in Women with Breast Cancer: A Systematic Review. J. Sci. Sport Exerc..

[68] Ordóñez-Mena J.M., Schöttker B., Fedirko V., Jenab M., Olsen A., Halkjær J., Kampman E., de Groot L., Jansen E., Bueno-de-Mesquita H.B. (2016). Pre-diagnostic vitamin D concentrations and cancer risks in older individuals: An analysis of cohorts participating in the CHANCES consortium. Eur. J. Epidemiol..

[69] Middleton P.G., Cullup H., Dickinson A.M., Norden J., Jackson G.H., Taylor P.R., Cavet J. (2002). Vitamin D receptor gene polymorphism associates with graft-versus-host disease and survival in HLA-matched sibling allogeneic bone marrow transplantation. Bone Marrow Transplant..

[70] Yu W.D., Sun G., Li J., Xu J., Wang X. (2019). Mechanisms and therapeutic potentials of cancer immunotherapy in combination with radiotherapy and/or chemotherapy. Cancer Lett..

[71] Kaiser M., Semeraro M.D., Herrmann M., Absenger G., Gerger A., Renner W. (2021). Immune Aging and Immunotherapy in Cancer. Int. J. Mol. Sci..

[72] Pouliliou S., Nikolaidis C., Drosatos G. (2020). Current trends in cancer immunotherapy: A literature-mining analysis. Cancer Immunol. Immunother..

[73] Topalian S.L., Weiner G.J., Pardoll D.M. (2011). Cancer Immunotherapy Comes of Age. J. Clin. Oncol..

[74] Rui R., Zhou L., He S. (2023). Cancer immunotherapies: Advances and bottlenecks. Front. Immunol..

[75] Chun R.F., Liu P.T., Modlin R.L., Adams J.S., Hewison M. (2014). Impact of vitamin D on immune function: Lessons learned from genome-wide analysis. Front. Physiol..

[76] Charoenngam N., Holick M.F. (2020). Immunologic Effects of Vitamin D on Human Health and Disease. Nutrients.

[77] Feldman D., Krishnan A.V., Swami S., Giovannucci E., Feldman B.J. (2014). The role of vitamin D in reducing cancer risk and progression. Nat. Rev. Cancer..

[78] Martens P.J., Gysemans C., Verstuyf A., Mathieu A.C. (2020). Vitamin D’s Effect on Immune Function. Nutrients.

[79] Artusa P., White J.H. (2025). Vitamin D and its analogs in immune system regulation. Pharmacol. Rev..

[80] Sîrbe C., Rednic S., Grama A., Pop T.L. (2022). An Update on the Effects of Vitamin D on the Immune System and Autoimmune Diseases. Int. J. Mol. Sci..

[81] Das D., Karthik N., Taneja R. (2021). Crosstalk Between Inflammatory Signaling and Methylation in Cancer. Front. Cell Dev. Biol..

[82] Holick M.F. (2017). The vitamin D deficiency pandemic: Approaches for diagnosis, treatment and prevention. Rev. Endocr. Metab. Disord..

[83] Giulietti A., van Etten E., Overbergh L., Stoffels K., Bouillon R., Mathieu C. (2007). Monocytes from type 2 diabetic patients have a pro-inflammatory profile. 1,25-Dihydroxyvitamin D(3) works as anti-inflammatory. Diabetes Res. Clin. Pract..

[84] Stucci L.S., D’Oronzo S., Tucci M., Macerollo A., Ribero S., Spagnolo F., Marra E., Picasso V., Orgiano L., Marconcini R. (2018). Italian Melanoma Intergroup (IMI). Vitamin D in melanoma: Controversies and potential role in combination with immune check-point inhibitors. Cancer Treat. Rev..

[85] Bersanelli M., Cortellini A., Leonetti A., Parisi A., Tiseo M., Bordi P., Michiara M., Bui S., Cosenza A., Ferri L. (2023). Systematic vitamin D supplementation is associated with improved outcomes and reduced thyroid adverse events in patients with cancer treated with immune checkpoint inhibitors: Results from the prospective PROVIDENCE study. Cancer Immunol. Immunother..

[86] Jeffery L.E., Burke F., Mura M., Zheng Y., Qureshi O.S., Hewison M., Walker L.S., Lammas D.A., Raza K., Sansom D.M. (2009). 1,25-Dihydroxyvitamin D3 and IL-2 combine to inhibit T cell production of inflammatory cytokines and promote development of regulatory T cells expressing CTLA-4 and FoxP3. J. Immunol..

[87] Adorini L., Daniel K.C., Penna G. (2006). Vitamin D receptor agonists, cancer and the immune system: An intricate relationship. Curr. Top. Med. Chem..

[88] Wang J., Zhu N., Su X., Gao Y., Yang R. (2023). Gut-Microbiota-Derived Metabolites Maintain Gut and Systemic Immune Homeostasis. Cells.

[89] Fan Y., Pedersen O. (2021). Gut microbiota in human metabolic health and disease. Nat. Rev. Microbiol..

[90] Adak A., Khan M.R. (2019). An insight into gut microbiota and its functionalities. Cell Mol. Life Sci..

[91] Zmora N., Suez J., Elinav E. (2019). You are what you eat: Diet, health and the gut microbiota. Nat. Rev. Gastroenterol. Hepatol..

[92] Liao X., Lan Y., Wang W., Zhang J., Shao R., Yin Z., Gudmundsson G.H., Bergman P., Mai K., Ai Q. (2023). Vitamin D influences gut microbiota and acetate production in zebrafish (*Danio rerio*) to promote intestinal immunity against invading pathogens. Gut Microbes.

[93] Góralczyk-Bińkowska A., Szmajda-Krygier D., Kozłowska E. (2022). The Microbiota-Gut-Brain Axis in Psychiatric Disorders. Int. J. Mol. Sci..

[94] Wang Z., Wang Z., Lu T., Chen W., Yan W., Yuan K., Shi L., Liu X., Zhou X., Shi J. (2022). The microbiota gut- brain axis in sleep disorders. Sleep Med. Rev..

[95] Zuo W.F., Pang Q., Yao L.P., Zhang Y., Peng C., Huang W., Han B. (2023). Gut microbiota: A magical multifunctional target regulated by medicine food homology species. J. Adv. Res..

[96] Sun J., Zhang Y.G. (2022). Vitamin D Receptor Influences Intestinal Barriers in Health and Disease. Cells.

[97] Assa A., Vong L., Pinnell L.J., Avitzur N., Johnson-Henry K.C., Sherman P.M. (2014). Vitamin D deficiency promotes epithelial barrier dysfunction and intestinal inflammation. J. Infect. Dis..

[98] Jin D., Wu S., Zhang Y.G., Lu R., Xia Y., Dong H., Sun J. (2015). Lack of Vitamin D Receptor Causes Dysbiosis and Changes the Functions of the Murine Intestinal Microbiome. Clin. Ther..

[99] Wang H., Tian G., Pei Z., Yu X., Wang Y., Xu F., Zhao J., Lu S., Lu W. (2025). *Bifidobacterium longum* increases serum vitamin D metabolite levels and modulates intestinal flora to alleviate osteoporosis in mice. mSphere.

[100] Yang X., Zhu Q., Zhang L., Pei Y., Xu X., Liu X., Lu G., Pan J., Wang Y. (2022). Causal relationship between gut microbiota and serum vitamin D: Evidence from genetic correlation and Mendelian randomization study. Eur. J. Clin. Nutr..

[101] Ma C., Han M., Heinrich B., Fu Q., Zhang Q., Sandhu M., Agdashian D., Terabe M., Berzofsky J.A., Fako V. (2018). Gut microbiome-mediated bile acid metabolism regulates liver cancer via NKT cells. Science.

[102] Song X., Sun X., Oh S.F., Wu M., Zhang Y., Zheng W., Geva-Zatorsky N., Jupp R., Mathis D., Benoist C. (2020). Microbial bile acid metabolites modulate gut RORγ^+^ regulatory T cell homeostasis. Nature.

[103] Izquierdo J.M. (2024). Vitamin D-dependent microbiota-enhancing tumor immunotherapy. Cell Mol. Immunol..

[104] Franco F., McCoy K.D. (2024). Microbes and vitamin D aid immunotherapy. Science.

